# Comparison of the selective targeting efficacy of *Salmonella typhimurium* A1-R and VNP20009 on the Lewis lung carcinoma in nude mice

**DOI:** 10.18632/oncotarget.3342

**Published:** 2015-01-21

**Authors:** Yong Zhang, Nan Zhang, Ming Zhao, Robert M. Hoffman

**Affiliations:** ^1^ AntiCancer, Inc., San Diego, California, USA; ^2^ Department of Surgery, University of California, San Diego, California, USA

**Keywords:** lung cancer, Salmonella typhimurium A1-R, VNP20009, GFP, RFP

## Abstract

*Salmonella typhimurium* A1-R is auxotrophic for arg and leu, which attenuates growth in normal tissue but allows high tumor targeting and virulence. A1-R is effective against metastatic human prostate, breast, and pancreatic cancer as well as osteosarcoma, fibrosarcoma, and glioma in clinically-relevant mouse models. VNP20009 is also a genetically-modified strain of *Salmonella typhimurium* that has been tested in Phase I clinical trials, but is more attenuated than *S. typhimurium* A1-R and in addition of multiple amino-acid auxotrophs, is purine auxotropic with the *purI* mutation. In the present study, mouse Lewis lung carcinoma-bearing nude mouse models were treated with *S. typhimurium* A1-R or VNP20009. *S. typhimurium* A1-R and VNP20009 were both eliminated from the liver and spleen approximately 3-5 days after administration via the tail vein. However, A1-R showed higher tumor targeting and inhibited the Lewis lung carcinoma to a greater extent than VNP20009, with less body weight loss. The mice tolerated *S. typhimurium* A1-R to at a least 2-fold higher dose than VNP20009 when the bacteria were administered iv. The results of the present study suggest that *S. typhimurium* A1-R has greater clinical potential than VNP20009.

## INTRODUCTION

For more than 200 years, cancers have been observed to regress following acute infections, mostly streptococcal [1]. In the late 19th and early 20th centuries. Coley infected cancer patients with *Streptococcus pyrogenes* and later treated the patients with extracts of the bacteria, which became known as Coley's toxins. Coley had remarkable results with both the bacteria and the toxins [2]. However bacterial therapy of cancer stopped after Coley's death in 1936 [2].

Recently, there has been intense renewed interest to develop bacterial therapy of cancer [2-4]. The barriers in tumors for standard therapy such as hypoxia, acidic pH, disorganized vascular architecture, are beneficial for bacteria to target cancer [3].

One approach to bacterial therapy of cancer is to use anaerobic bacteria such as *Bifidobacterium* [5] and *Clostridium* [6] which replicate in necrotic areas of tumors. These anaerobic bacteria cannot grow in viable tumor tissue, which restricts their efficacy. In addition, obligate anaerobic bacteria may be limited to intratumor injection which would preclude their use for metastatic cancer.

Recently a human patient with metastatic leiomyosarcoma was treated by intratumoral injection of *Clostridium novyi* (*C. novyi*)-NT spores which reduced the tumor within and surrounding the bone [7]. However other tumor deposits in the patient were not affected.

*S. typhimurium* is a facultative anaerobe and therefore unlike anaerobe bacteria can infect viable portions of tumors as well as necrotic areas. The VNP20009 strain of *S. typhimurium*, attenuated with purine and other auxotrophic mutations, has been previously used for cancer therapy [8]. When NP20009 were inoculated i.p. into C57B6 mice bearing the B16F10 melanoma, it suppressed tumor growth and prolonged average survival to as much as twice that of untreated mice [9].

VNP20009 is attenuated with a lipid A–mutation (*msbB*), purine auxotrophy (*purI*) and amino acid auxotrophs [8]. In a Phase I clinical trial on patients with metastatic melanoma and renal-cell carcinoma, VNP20009 was safely administered, but poorly colonized the patients' tumors, perhaps because it was overattenuated [10]. At the highest tolerated dose, some tumor colonization was observed [10].

Another strain of *S. typhimurium*, A1-R, has been developed by our laboratory, which has greatly increased antitumor efficacy. *S. typhimurium* A1-R is auxotrophic only for leu-arg which prevents it from mounting a continuous infection in normal tissues. *S. typhimurium* A1-R has no other attenuating mutations as does VNP20009. A1-R was able to eradicate primary and metastatic tumors in monotherapy in nude mouse models of prostate, breast, ovarian and pancreatic cancer, as well as sarcoma and glioma [11-19]. *S. typhimurium* A1-R also greatly inhibited bone and brain metastasis of breast cancer in orthotopic mouse models [20, 21]. Tumors with a high degree of vascularity were more sensitive to *S. typhimurium* A1-R, and vascular destruction appears to play a role in *S. typhimurium* A1-R antitumor efficacy [22].

The present study compares *S. typhimurium* A1-R and VNP20009 for anti-tumor efficacy in a nude mouse model of highly aggressive lung cancer.

## RESULTS AND DISCUSSION

### Comparison of toxicity of *S. typhimurium* A1-R and VNP20009

**Figure 1:**
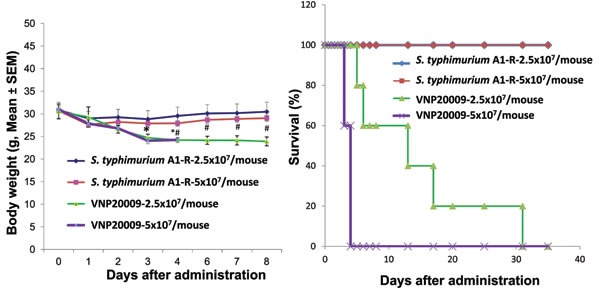
Comparison of body weight and survival of nude mice without tumors after *S. typhimurium* A1-R or VNP20009 i.v. administration Female nude mice, aged 6 weeks, were injected iv with *S. typhimurium* A1-R or VNP20009 at different doses in 100 μl PBS. Body weight was monitored using an electronic scale. A: Body weight curve comparing *S. typhimurium* A1-R and VNP20009 at both doses (*p*<0.05). B: Kaplan Meir survival curve. n = 5 mice for each group comparing *S. typhimurium* A1-R and VNP20009 at both doses (*p*<0.05)

There was lower toxicity of *S. typhimurium* A-1R in nude mice compared to *S. typhimurium* VNP20009. Treatment with *S. typhimurium* A1-R resulted in less body weight loss than with VNP20009 (*p* = <0.05) (Figure 1A). There was prolonged survival in mice treated with *S. typhimurium* A1-R as compared to the non-tumor-bearing mice treated with *S. typhimurium* VNP20009 (*p = 0.005*) (Figure 1B).

**Figure 2:**
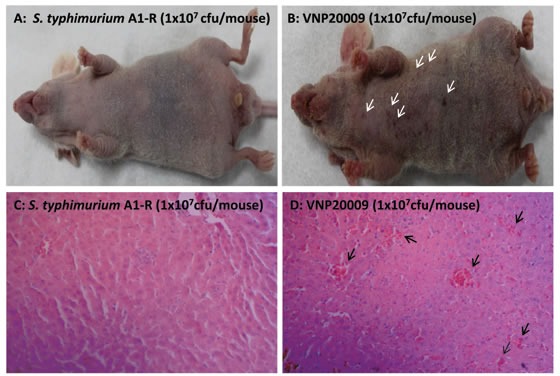
Comparison of overt toxicity of *S. typhimurium* A1-R and VNP20009 A: *S. typhimurium* A1-R treated mice had few hemorrhagic spots on the skin. B: VNP20009 treated mice had more hemorrhagic spots on the skin (white arrows). C: *S. typhimurium* A1-R treated mice had less bleeding foci on the liver on day 3 after bacteria infection. D: VNP20009 treated mice had more bleeding foci on the liver on the day 3 than *S. typhimurium* A1-R after bacteria infection (black arrows) (*p*<0.05)

There were less hemorrhagic spots on the skin and liver in mice treated with *S. typhimurium* A1-R than VNP20009 (Figure 2). Bleeding foci were found in the liver on day 3 after bacteria injection. However, VNP20009 has more bleeding foci on the liver than in *S. typhimurium* A1-R-treated mice (*p* <0.05).

### Comparison of distribution of *S. typhimurium* A1-R and VNP20009 in tumor, liver, spleen, and blood

**Figure 3:**
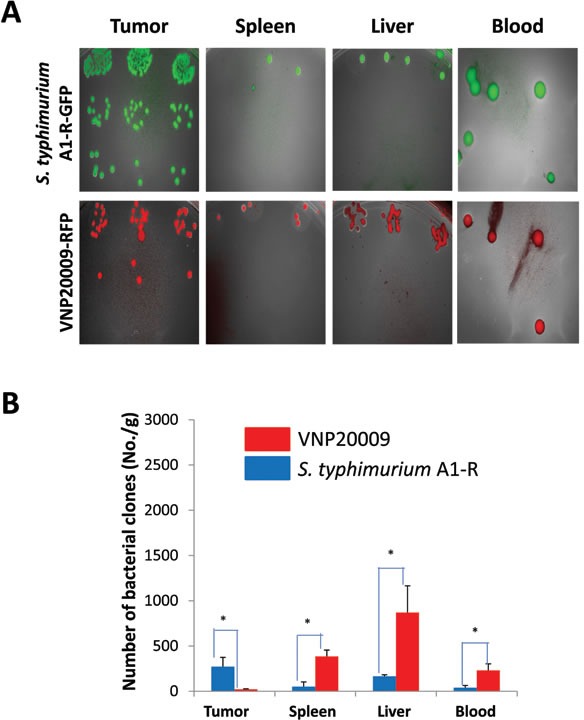
Comparison of tissue distribution of *S. typhimurium* A1-R and VNP20009 in tumor and normal tissues When the average tumor volume reached approximately 70 mm^3^, *S. typhimurium* A1-R (1×10^7^ CFU) or VNP-20009 (1×10^7^ CFU) were injected into the tail vein. Tissues were removed 6 days after bacteria administration. Bacteria were isolated from the tumor and organs and cultured in LB agar (*p<0.05)

When the average tumor volume reached approximately 70 mm^3^, *S. typhimurium* A1-R (1×10^7^ CFU) or VNP-20009 (1×10^7^ CFU) were injected into the tail vein one time. Tissues were removed 6 days after bacteria administration. Bacteria were isolated from the tumor and organs and cultured in LB agar. Both strains selectively targeted the tumor with greater targeting by *S. typhimurium* A1-R than VNP20009. VNP20009 had more bacteria in the liver and spleen and blood (*p* <0.05) (Figure 3).

### Comparison of efficacy of *S. typhimurium* A1-R and VNP20009

**Figure 4:**
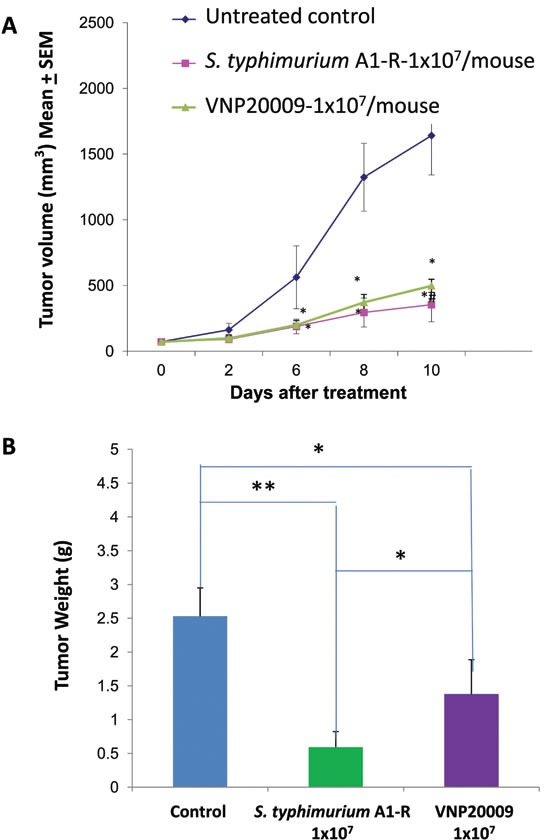
Comparison of efficacy of *S. typhimurium* A1-R or VNP20009 on the Lewis lung cancer When the average tumor volume reached approximately 70 mm^3^, *S. typhimurium* A1-R (1×10^7^ CFU) or VNP-20009 (1×10^7^ CFU) were injected into the tail vein once a week. Primary tumor sizes were measured with a caliper. A: Tumor growth curve. B: Tumor weight in *S. typhimurium* A1-R- and VNP20009-treated mice, as compared to control mice (*p<0.05; **p<0.01) at day 10 after treatment. n = 5 mice for each group

*S. typhimurium* A1-R reduced tumor growth to a greater extent than VNP20009 (*p* < 0.05) (Figure 4A). On day 10, a significantly lower tumor burden in mice treated with *S. typhimurium* A1-R than mice treated with VNP20009 was observed. *S. typhimurium* A1-R-treated mice had a tumor weight (0.594 g ± 0.23) which was lower than with VNP20009-treated mice (1.378 g ± 0.51) (*p*<0.05). Control mice had a tumor weight of (2.53 g ± 0.42) which was greater compared to VNP20009-treated mice (*p* <0.05) or *S. typhimurium* A1-R-treated mice (p<0.01) (Figure 4B).

## CONCLUSIONS

*S. typhimurium* A1-R had lower toxicity in nude mice compared to *S. typhimurium* VNP20009. *S. typhimurium* A1-R and *S. typhimurium* VNP20009 both inhibited growth of the Lewis lung cancer in nude mouse subcutaneous models. However, *S. typhimurium* A1-R was more efficacious than *S. typhimurium* VNP20009 on tumor growth inhibition.

The results of the present study indicate *S. typhimurium* A1-R should enter clinical trials as soon as possible. Better results with *S. typhimurium* A1-R than VNP20009 should be expected since *S. typhimurium* A1-R is more virulent against the tumor and less toxic against the host. Better results with *S. typhimurium* A1-R than *C. novyi-*NT should be expected since *S. typhimurium* A1-R can be effectively administered systemically and *C. novyi-*NT seems limited to intratumor administration, possibly precluding it from targeting metastatic disease. A promising application of *S. typhimurium* A1-R is to decoy cancer cells within tumor to divide and thereby become sensitive to chemotherapy [23].

The bacterial tumor targeting described in the present report can take advantage of previously developed tumor targeting strategies [24-31].

## MATERIALS AND METHODS

### Cells

Lewis lung carcinoma cells were maintained in RPMI 1640 supplemented with 10% fetal bovine serum, 2 mM glutamine (Gibco-BRL, Life Technologies, Inc., Carlsbad, CA). The cell line was cultured at 37°C in a 5% incubator. RFP vector production and transduction of the Lewis lung cancer cell line were performed as previously published [32-34]. The RFP (DsRed-2) gene (Clontech Laboratories, Palo Alto, CA) was inserted in the retroviral-based mammalian expression vector pLNCX (Clontech) to form the pLNCX-DsRed-2 vector as previously described [32-34]. For RFP gene transduction, 70% confluent Lewis lung carcinoma cells were incubated with a 1:1 precipitated mixture of pLNCX DsRed-2-retroviral-containing supernatants of PT67 packaging cells and RPMI 1640 or other culture medium (Life Technologies) containing 10% fetal bovine serum (Atlanta Biologicals, Norcross, GA) for 72 h and selected using G418 [35].

### Mice

Nude mice were from AntiCancer Inc. (San Diego, CA). All mouse studies were conducted with an AntiCancer Institutional Animal Care and Use Committee (IACUC)-protocol specifically approved for this study and in accordance with the principals and procedures outlined in the National Institute of Health Guide for the Care and Use of Animals under Assurance Number A3873-1. The animals were observed on a daily basis and humanely sacrificed by CO_2_ inhalation when they met the following humane endpoint criteria: prostration, skin lesions, significant body weight loss, difficulty breathing, epistaxis, rotational motion and body temperature drop. Animals were housed in a barrier facility on a high efficiency particulate air (HEPA)-filtered rack under standard conditions of 12-hour light/ dark cycles. Animals were housed with no more than 5 per cage. The animals were fed an autoclaved laboratory rodent diet.

### Mouse model of aggressive lung cancer

Mouse tumor models were made by subcutaneously injecting Lewis lung carcinoma cells expressing RFP (LLC-RFP) cells (3 × 10^6^ cells/100 μl PBS) in the flank of nude mice [32].

### Preparation of *S. typhimurium* A1-R and VNP20009

Green fluorescent protein (GFP)-expressing *Salmonella typhimurium* A1-R bacteria and RFP-labeled VNP20009 were grown overnight in LB medium and then diluted 1:10 in LB medium, respectively. Bacteria were harvested at late-log phase, washed with PBS, and then diluted in PBS.

### Comparison of toxicity of *S. typhimurium* A1-R and VNP20009

Twenty nude mice, 6 weeks old, were used to compare toxicity of *S. typhimurium* A1-R and VNP20009. The nude mice were randomized into 4 groups of 5 mice. Group 1 mice were treated with *S. typhimurium* A1-R (2.5 × 10^7^cfu/100 μl i.v.), once a week for 5 weeks. Group 2 mice were treated with *S. typhimurium* A1-R (5 × 10^7^cfu/100 μl i.v.), once a week for 5 weeks. Group 3 mice were treated with *S. typhimurium* VNP20009 (2.5 × 10^7^cfu/100 μL i.v.), once a week for 5 weeks. Group 4 mice were treated VNP20009 (5 × 10^7^cfu/100 μL i.v.), once a week for 5 weeks. All mice were used for survival determination for 35 days post initial treatment.

### Efficacy experiments

Group 1 mice served as untreated controls. Ten mice in group 2 were treated with *S. typhimurium* A1-R (1 × 10^7^cfu/100 μl i.v.), once a week for 2 weeks. Ten mice in group 3 were treated iv with VNP20009 (1 × 10^7^cfu/100 μl i.v.), once a week for 2 weeks. On the 3^rd^ day after treatment, five mice in each group were sacrificed. Bacteria were isolated from tumors, the liver, spleen and blood and cultured in LB agar. For the remaining five mice in each group, the tumors were removed by surgery to compare with the control group on day 10 after treatment. Tumor weight was determined on day 10.

### Imaging

Bacteria from the organs were imaged with the Maestro fluorescence imaging system (Perkin-Elmer Inc.). The Maestro multispectral imaging systems contains a liquid crystal tunable filter (LCTF) optically coupled to a CCD camera. Multispectral images are acquired with images typically spaced every 10 nm throughout the desired spectral rang [36-38].
